# Establishment and verification of the nomogram that predicts the 3-year recurrence risk of epithelial ovarian carcinoma

**DOI:** 10.1186/s12885-020-07402-2

**Published:** 2020-09-29

**Authors:** Jun Hu, Xiaobing Jiao, Lirong Zhu, Hongyan Guo, Yumei Wu

**Affiliations:** 1 Department of Gynecology and Obstetrics, Peking University First Hospital, No.1Xi’anmen Street, Xicheng District, Beijing, 100034 China; 2 Department of Gynecology and Obstetrics, Peking University Third Hospital, No. 49 North Garden Road, Haidian District, Beijing, 100191 China; 3 Department of Gynecology and Obstetrics, Beijing Obstetrics and Gynecology Hospital, Capital Medical University, No. 251 Yaojiayuan Road, Chaoyang District, Beijing, 100026 China

**Keywords:** Ovarian epithelial carcinoma, Recurrence free interval, Recurrence risk, Nomograms, Verification

## Abstract

**Background:**

As we all know, patients with epithelial ovarian carcinoma have poor prognosis and high recurrence rate. It is critical and challenging to screen out the patients with high risk of recurrence. At present, there are some models predicting the overall survival of epithelial ovarian carcinoma, however, there is no widely accepted tool or applicable model predicting the recurrence risk of epithelial ovarian carcinoma patients. The objective of this study was to establish and verify a nomogram to predict the recurrence risk of EOC.

**Methods:**

We reviewed the clinicopathological and prognostic data of 193 patients with EOC who achieved clinical complete remission after cytoreductive surgery and chemotherapy between January 2003 and December 2013 in Peking University First Hospital. The nomogram was established with the risk factors selected by LASSO regression. The medical data of 187 EOC patients with 5-year standard follow-up in Peking University Third Hospital and Beijing Obstetrics and Gynecology Hospital were used for external validation of the nomogram. AUC curve and Hosmer-Lemeshow test were used to evaluate the discrimination and calibration.

**Results:**

The nomogram for 3-year recurrence risk was established with FIGO stage, histological grade, histological type, lymph node metastasis status and serum CA125 level at diagnosis. The total score can be obtained by adding the grading values of these factors together. The C statistics was 0.828 [95% CI, 0.764–0.884] and the Chi-square value is 3.6 (*P* = 0.731 > 0.05) with the training group. When the threshold value was set at 198, the sensitivity, specificity, positive predictive value, negative predictive value and concordance index were 88.8, 67.0, 71.8, 86.3% and 0.558 respectively. In the external validation, the C statistics was 0.803 [95%CI, 0.738–0.867] and the Chi-square value is 11.04 (*P* = 0.135 > 0.05). With the threshold value of 198, the sensitivity, specificity, positive predictive value, negative predictive value and concordance index of the nomogram were 75.7, 77.0, 83.2, 67.9%, and 0.52 respectively.

**Conclusions:**

We established and validated a nomogram to predict 3-year recurrence risk of patients with EOC who achieved clinical complete remission after cytoreductive surgery and chemotherapy. This nomogram with good discrimination and calibration might be useful for screening out the patients with high risk of recurrence.

## Background

Epithelial ovarian carcinoma is a common gynecological malignancy. 75% of the cases were diagnosed as advanced stage (stage III/IV), and the 5-year survival rate was only 20–39% [1, 2]. Even in the patients who have achieved clinical complete remission (CCR) after active treatment, 25% of the early stage (stage I / II) patients with epithelial ovarian cancer and 80% of advanced stage patients with epithelial ovarian cancer will eventually relapse [3, 4]. Although patients with primary early-stage ovarian cancer have an overall favorable prognosis, survival after recurrence is poor and comparable to those with recurrent advanced-stage disease [5]. It is critical and challenging to screen out the patients with high risk of recurrence. To predict the recurrence risk of patients with EOC, we need to combine clinicopathological factors, such as FIGO staging, histological grade, histological type, lymph node metastasis, carbohydrate antigen 125 (CA125) level. At present, there is no widely accepted tool or model predicting the recurrence risk of EOC patients. The purpose of this study is to identify the influencing factors of recurrence in patients with epithelial ovarian cancer by retrospective cohort study, and to establish a nomogram for predicting recurrence risk, so as to provide a convenient quantitative standard for clinical treatment of patients with EOC and for judging recurrence risk.

## Methods

### Study population

**Fig. 1:**
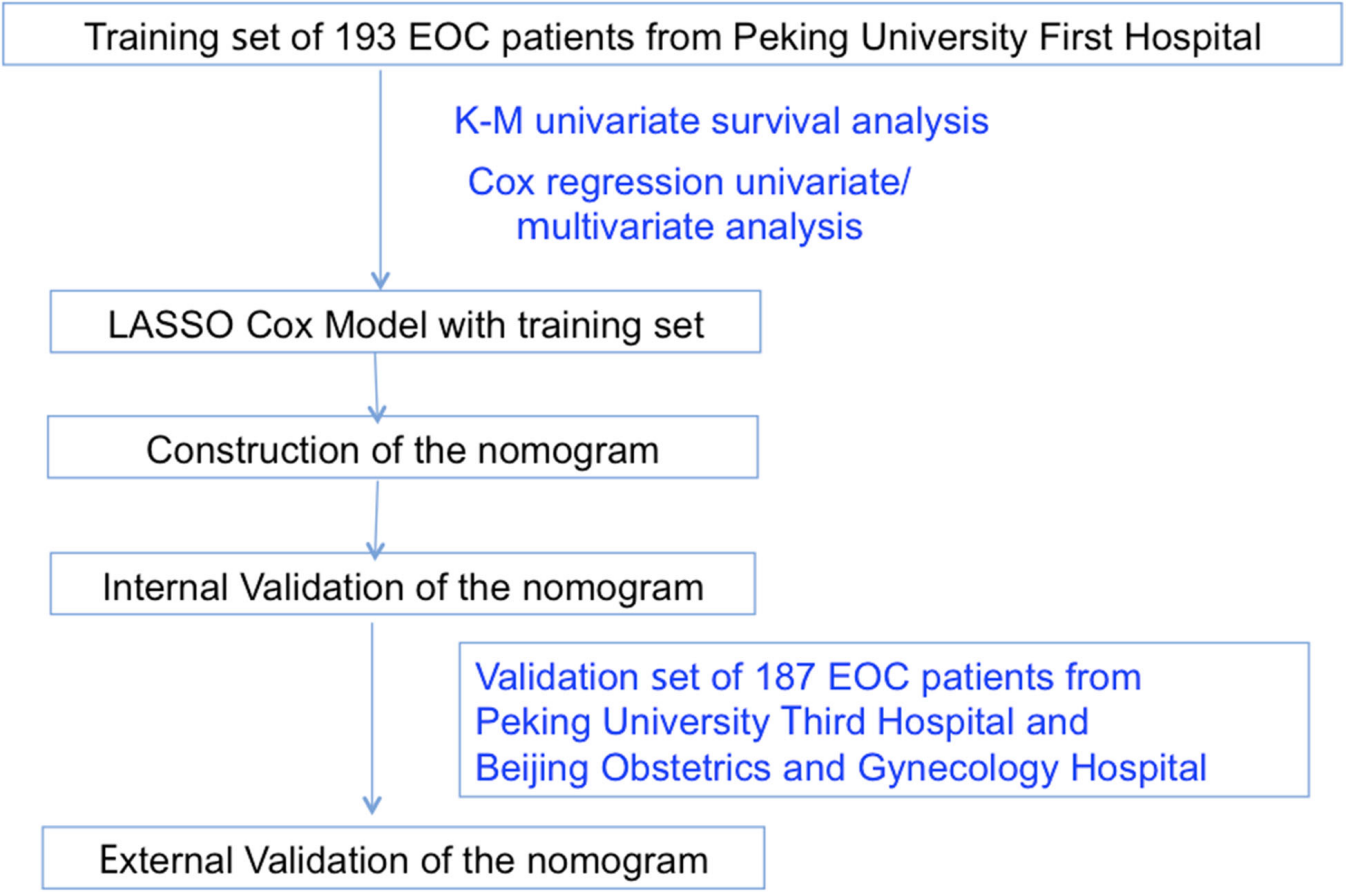
The flow chart of the study

The patients diagnosed as EOC were enrolled from Peking University First Hospital, Peking University Third Hospital and Beijing Obstetrics and Gynecology Hospital between January 2003 and December 2013. The inclusion criteria were EOC patients who reached CCR after initial or intermediate cytoreductive surgery and standard adjuvant platinum-based chemotherapy. Patients who received fertility sparing surgery or with history of other malignant tumors were excluded. CCR is defined as: (1) the level of serum CA125 is within the normal range; and (2) no residual lesions are found by imaging examination after primary treatments. General information, size of residual lesions, FIGO stage, histological grade, histological type, lymph node metastasis, expression of estrogen receptor (ER), progesterone receptor (PR) and Ki67, adjuvant therapy and serum CA125 level were collected from the original medical records. All the patients were followed up by telephone call and clinical visits. Follow-up was conducted every 2–4 months in the first and second year, every 3–6 months in the third, fourth and fifth year, and every year after 5 years. All patients were followed up until 30 June 2019. The end points of follow-up were recurrence, no recurrence but death, or no recurrence and no death at the end of observation. The definition of recurrence of EOC is that the serum CA125 level is higher than the normal value (35 U/mL) and/or the recurrence focus is found by imaging examination. Recurrence-free interval (RFI) is defined as the interval between the recurrence and the end of last chemotherapy of first line treatment. The EOC patients who fulfilled the criteria from Peking University First Hospital were enrolled into the training group to establish the nomogram. And the medical data of EOC patients with 5-year standard follow-up in Peking University Third Hospital and Beijing Obstetrics and Gynecology Hospital were used for external validation of the nomogram. The flow chart of the study was shown in Fig. 1. This study was approved by the Ethics Committee of the First Hospital of Peking University (Scientific Research No. 2018–109).

### Prognostic models

Kaplan-Meier univariate survival analysis, Log-rank test and Cox univariate and multivariate regression analysis were used to screen out the factors related to recurrence in patients with EOC. Least absolute shrinkage and selection operator regression was used to analyze the related factors. The 3-year recurrence rate nomogram was established with the risk factors selected by LASSO regression. Bootstrap resampling, AUC curve and Hosmer-Lemeshow good of fit test were used to evaluate the discrimination and calibration.

### External validation/statistical analysis

Recurrence probabilities were calculated using the nomograms for every patient in the validation set. 3-year recurrence rate were obtained using the method of Kaplan–Meier. The discriminative ability was measured with the c index. Calibration was assessed graphically by means of the R package rms. The software used for statistical analysis includes SPSS 23.0, R 3.5.2 and EmpowerStats. Differences were considered to be significant at *P* < 0.05.

## Results

### Validation cohorts

One hundred ninety-three EOC patients from Peking University First Hospital were enrolled into the training group. The characteristics of these patients, including age, FIGO stage, histological grade, histological type, lymph node metastasis, residual lesion size, serum CA125 level and molecular markers of tumor tissues were summarized in Table 1. One hundred six cases (54.9%) had recurrence. The RFI ranged from 1.8 months to 173.2 months, with a median of 46.7 months. Seventy-seven cases had no recurrence; 10 cases had censored data, 9 cases had lost follow-up, 1 case died of other disease, the rate of lost follow-up was 4.7%. The results of Kaplan Meier survival analysis and log rank test are summarized in Table 1.

**Table 1:** Kaplan-Meier single factor survival analysis of patients in training group

Factors	Stratification factor	Number(%)	Median RFI (months)	*P* value
Age	≤50 years old	68 (35.2%)	53.0	0.778
	> 50 years old	125 (64.8%)	48.0	
FIGO stage	I	55 (28.5%)	NA	< 0.001
	II	26 (13.5%)	NA	
	III	92 (47.7%)	18.0	
	IV	20 (10.4%)	10.1	
Histological grade	G1	37 (19.2%)	NA	< 0.001
	G2	45 (23.3%)	27.6	
	G3	111 (57.5%)	26.4	
Histological type	Serous carcinoma	117 (60.6%)	24.0	< 0.001
	Non-serous carcinoma*	76 (39.4%)	NA	
Postoperative residual size	0	78 (40.4%)	NA	< 0.001
	< 1 cm	88 (45.6%)	26.8	
	≥1 cm	27 (14.0%)	18.0	
Lymph node status	No metastasis	53 (27.5%)	NA	< 0.001
	Metastasis	17 (8.8%)	10.1	
	No Lymphonectomy	123 (63.7%)	27.3	
Pretreatment CA125 level	< 35 U/mL	30 (15.5%)	NA	< 0.001
	≥35 U/mL and < 1000 U/mL	118 (61.1%)	53.0	
	≥1000 U/mL	45 (23.3%)	16.1	
Expression of ER in tumor tissues	Negative	70 (36.3%)	NA	0.008
	Positive	123 (63.7%)	32.5	
Expression of PR in tumor tissues	Negative	81 (42.0%)	27.6	0.192
	Positive	112 (58.0%)	94.5	

Non-serous cancers include endometrioid, clear cell, mucinous, undifferentiated and mixed epithelial tumors.

*NA* Not available.

Cox regression univariate analysis showed that FIGO staging, histological grade, histological type, size of residual lesions after surgery, lymph node metastasis, pre-treatment CA125 level, ER expression in tumor tissue had significant differences in the impact of internal stratification on recurrence. Cox regression multivariate analysis showed that advanced EOC, histological grade and histological type were independent risk factors for recurrence of epithelial ovarian cancer. The results of specific stratification factor were shown in Table 2.

**Table 2:** The result of Cox multi-regression survival analysis

Factors	Stratification factor	HR	95%CI	*P*
FIGO stage	I	1		
	II	2.3	0.8–6.4	0.102
	III	5.9	2.1–16.4	0.001
	IV	6.3	2.0–20.0	0.002
Histological grade	G1	1		
	G2	6.4	1.4–28.4	0.015
	G3	6.9	1.6–31.1	0.011
Histological type	Serous carcinoma	1		
	Non-serous carcinoma^*^	1.8	1.1–2.9	0.027
Postoperative residual size	0	1		
	< 1 cm	0.6	0.3–1.1	0.099
	≥1 cm	0.7	0.4–1.5	0.392
Lymph node status	No metastasis	1		
	Metastasis	2.0	0.8–4.8	0.114
	Not available	1.6	0.8–3.3	0.158
Pretreatment CA125 level	< 35 U/mL	1		
	≥35 U/mL and < 1000 U/mL	1.7	0.6–5.0	0.304
	≥1000 U/mL	2.0	0.7–6.2	0.210
Expression of ER in tumor tissues	Negative	1		
	Positive	1.0	0.6–1.6	0.942

**Fig. 2:**
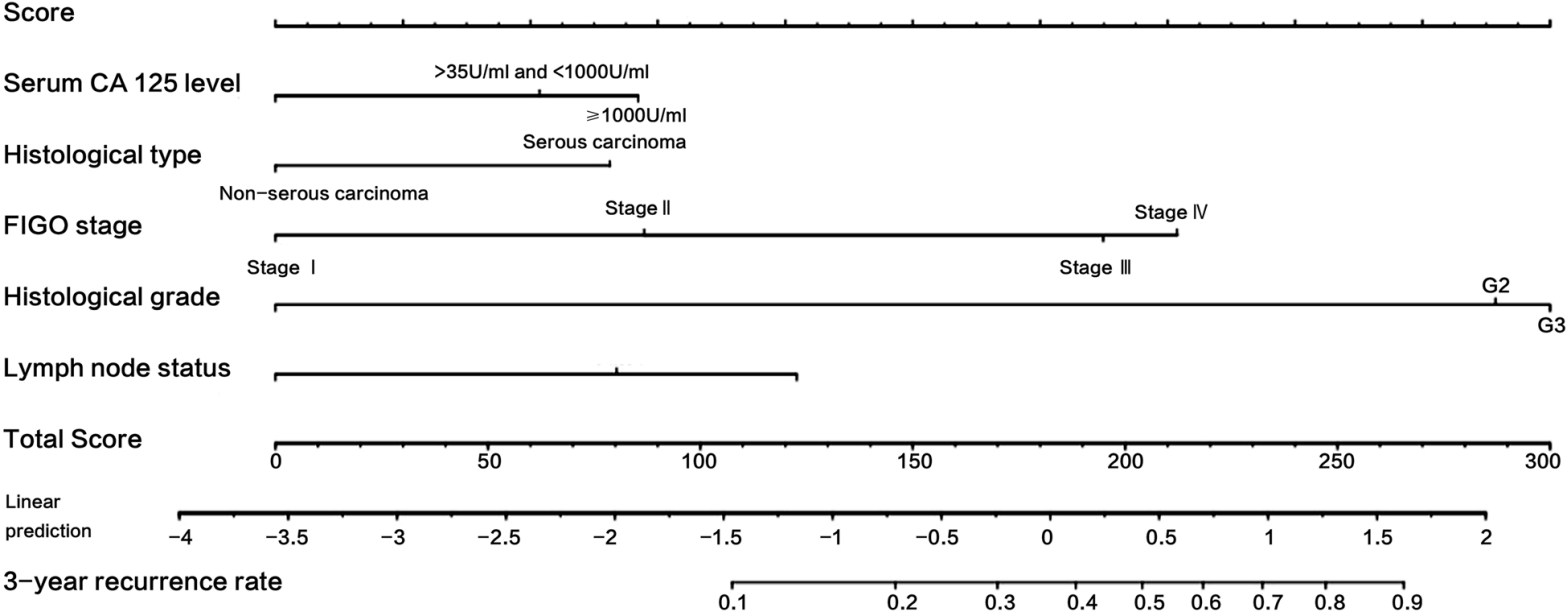
A nomogram for predicting 3-year recurrence risk in EOC patients. Note: A line perpendicular to and intersecting with the grading coordinate axis is drawn upward from the position of the grading factors of each influencing factor coordinate axis. When the grading values of the five influencing factors are determined, the total score can be obtained by adding them together. Draw a line perpendicular to and intersecting with the coordinate axis of predicting recurrence rate from the position of total score. The intersection point is the 3-year predicted recurrence rate related to the total score

LASSO regression was used to screen the best influencing factors for the establishment of the model. The optimal number of factors used to establish the contour map prediction model was 5. The final selected model included the following 5 variables: FIGO staging, histological grade, histological type, lymph node metastasis and serum CA125 level before treatment. Each stratification factor is assigned with a specific grading value (see Table 3 for details). When the grading values of the five influencing factors are determined, the total score can be obtained by adding them together. Figure 2 showed the nomogram for predicting 3-year recurrence risks of patients with EOC. The mathematical formulas between the total score and the recurrence rate for 3 years are as follows:

$$ 3-\mathrm{year}\ \mathrm{recurrence}\ \mathrm{rate}=1-\left[1.51\mathrm{e}-07\ast \mathrm{total}\ \mathrm{score}\hat{\mkern6mu} 3+\left(-0.000101727\right)\ast \mathrm{total}\ \mathrm{score}\hat{\mkern6mu} 2+0.016191444\ast \mathrm{total}\ \mathrm{score}+0.144929485\right] $$

**Table 3:** Scores for Recurrence related Factors

Recurrence related Factors	Stratification factor	Score
FIGO stage	I	0
	II	29
	III	65
	IV	71
Histological grade	G1	0
	G2	96
	G3	100
Histological type	Serous carcinoma	0
	Non-serous carcinoma	26
Lymph node status	No metastasis	0
	Metastasis	41
	Not available	27
Pretreatment CA125 level	< 35 U/mL	0
	≥35 U/mL and < 1000 U/mL	21
	≥1000 U/mL	28

For example, a patient with EOC had a serum CA125 level of 600 U/ml (21 points) underwent the initial cytoreductive surgery. Pathology result showed that she was stage IIIC (65 points), serous carcinoma (26 points), grade G3 (100 points), lymph node metastasis (41 points) and she has reached CCR after 6 cycles of standardized chemotherapy. According to the above-mentioned nomogram, the total score of the patient was 253. The relatively overall 3-year predicted recurrence rate for this patient was 82.01%.

**Fig. 3:**
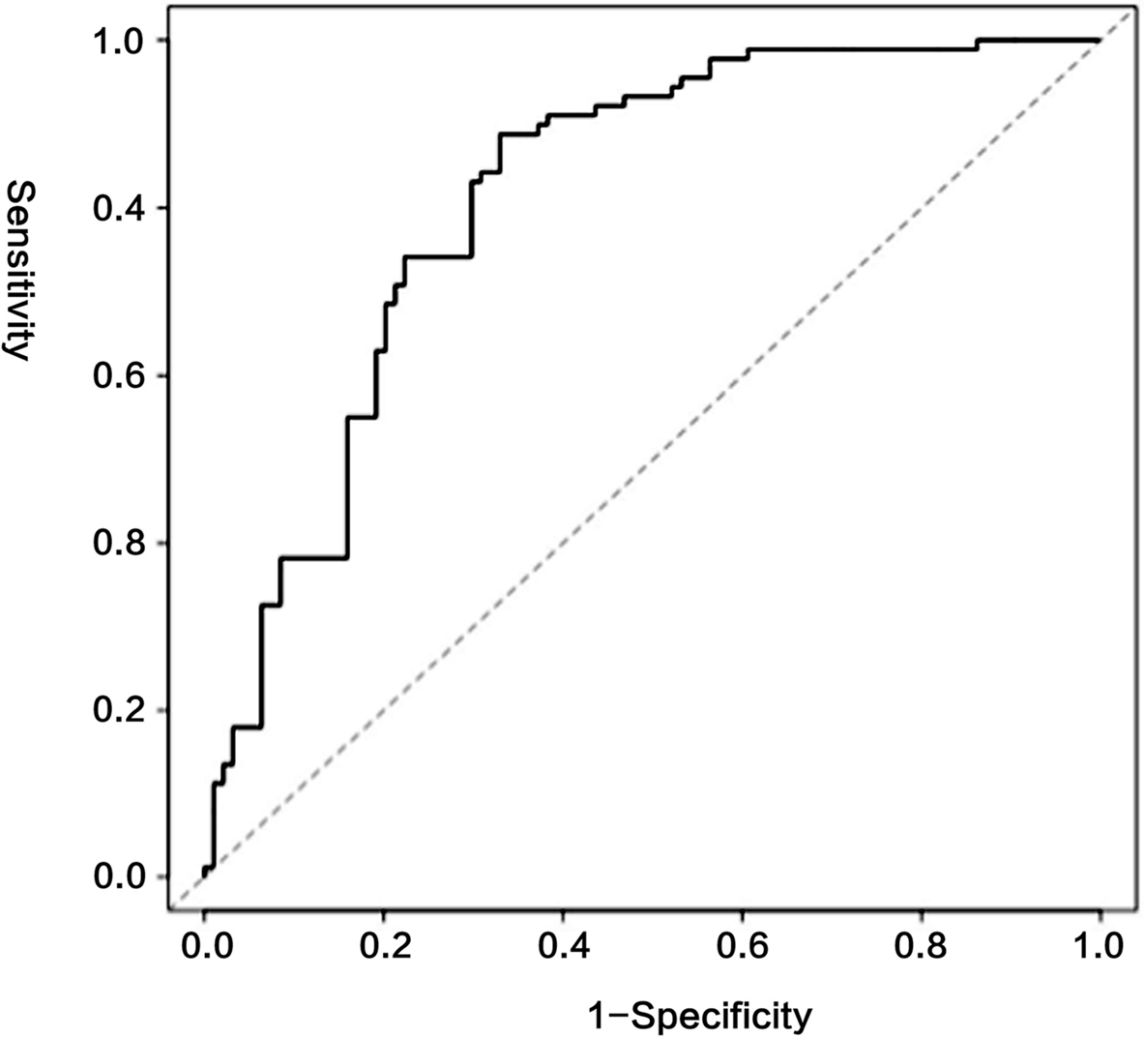
ROC curve of the nomogram with the training group

**Fig. 4:**
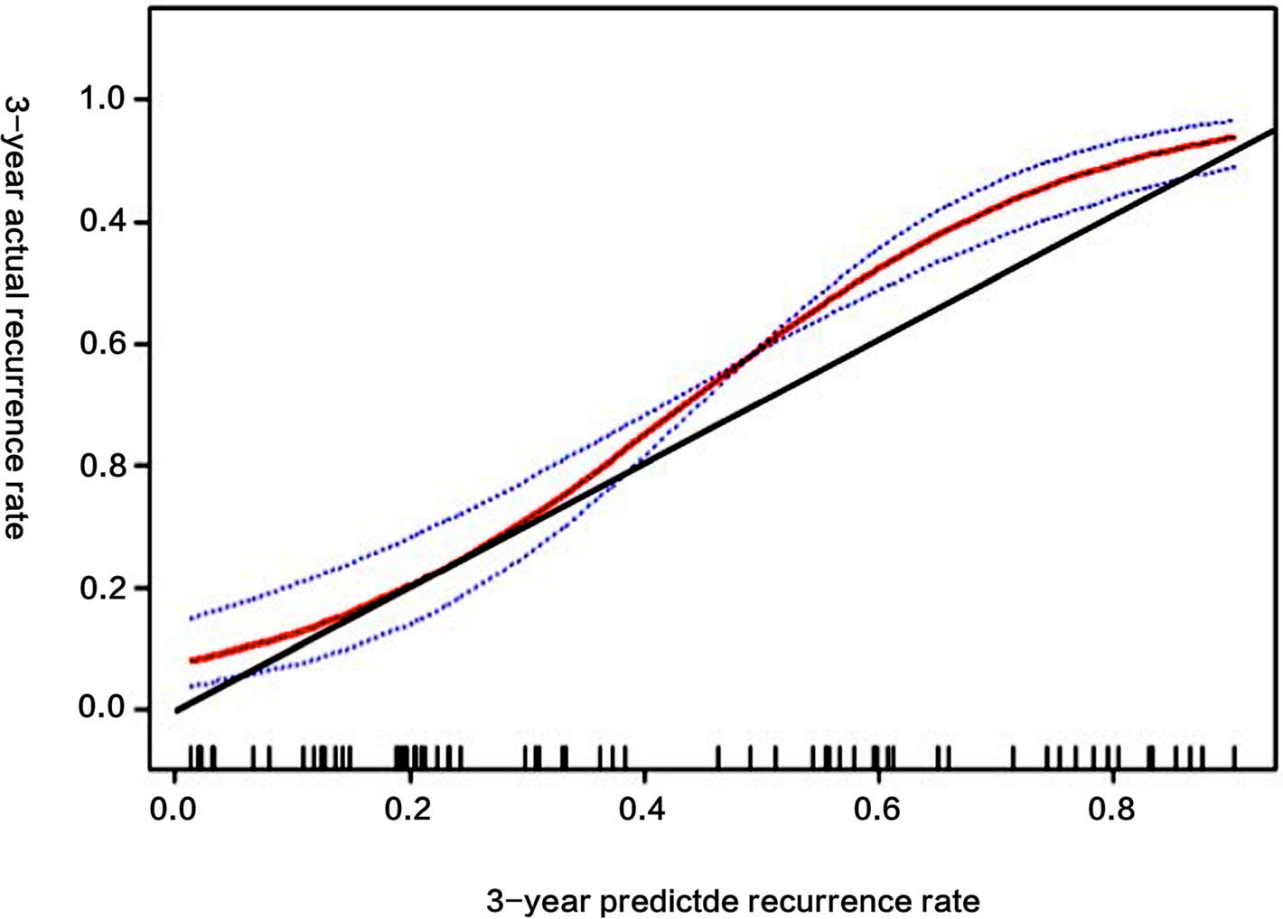
The calibration curve of the nomogram. Note: The horizontal coordinate axis of the chart is a 3-year predicted recurrence rate, and the vertical coordinate axis is a 3-year actual recurrence rate. The red curve is a calibration curve which corresponds to the actual recurrence rate. The blue curve represents the 95% CI range of the calibration curve. The black line is an ideal calibration when the 3-year predicted recurrence rate is equal to the actual recurrence rate

The ROC curve of the nomogram with internal validation was shown in Fig. 3. The AUC (C statistics) was 0.828 (95% CI, 0.764–0.884). When the threshold value was set at 198, the sensitivity, specificity, positive predictive value, negative predictive value and concordance index were 88.8, 67.0, 71.8, 86.3% and 0.558 respectively. Patients with total score higher than 198 were identified with high-risk recurrence and those with total score lower than 198 were identified with low-risk recurrence. Hosmer-Lemeshow test for evaluation of calibration showed that the Chi-square value is 3.6 (*P* = 0.731 > 0.05), As the calibration curve shown in Fig. 4, if the 3-year predicted recurrence rate calculated by the model is within the range of 15 to 30%, the predicted value is basically consistent with the actual recurrence rate; if the predicted value is below 15% or above 30%, the predicted value is less than the actual recurrence rate, indicating that the recurrence risk is underestimated.

### External validation

**Fig. 5:**
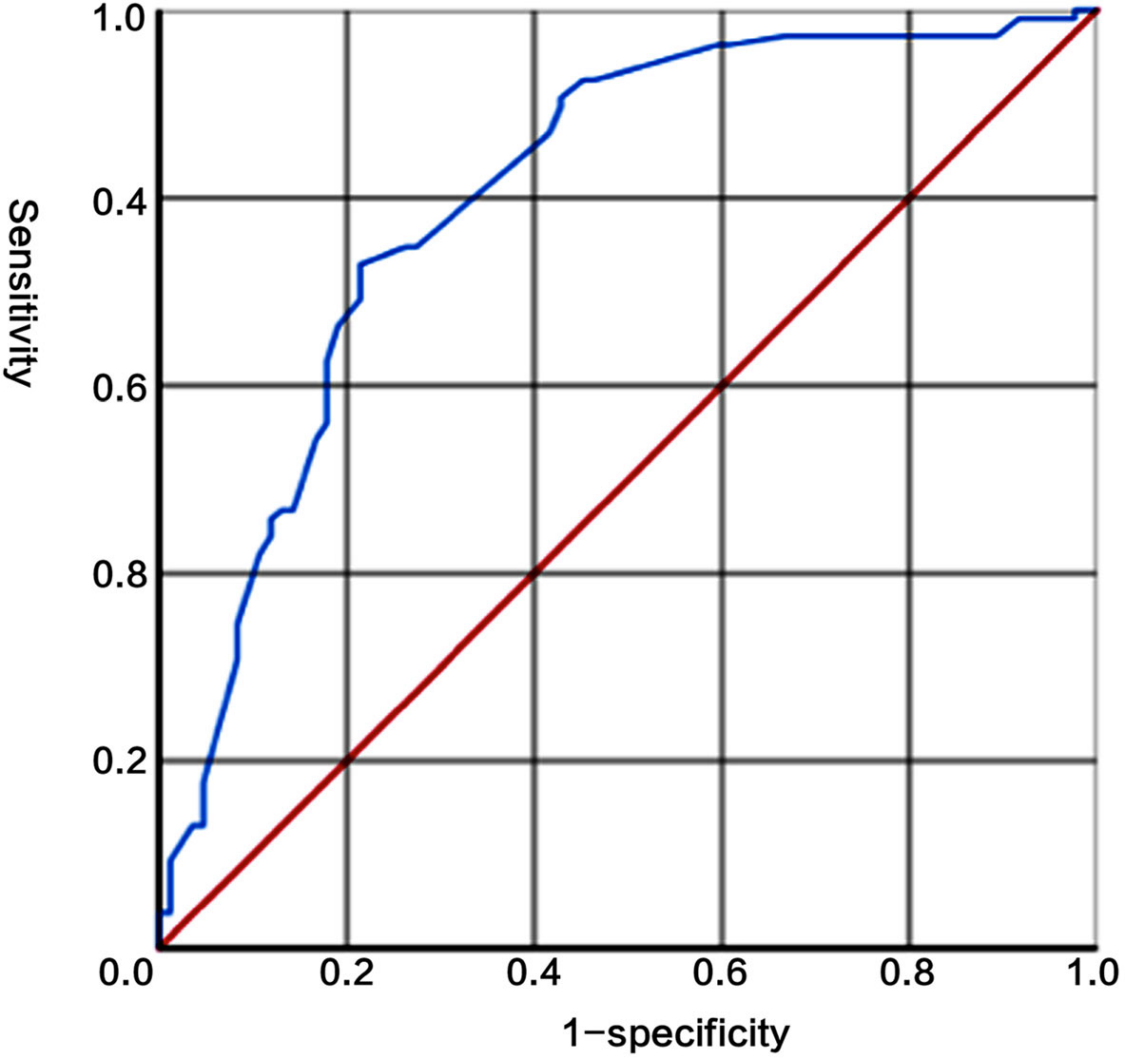
ROC curve of the nomogram with the external verification group

The medical data of 187 EOC patients from in Peking University Third Hospital and Beijing Obstetrics and Gynecology Hospital were enrolled into the external validation group. The ROC curve of the nomogram with external validation was shown in Fig. 5. The AUC (C statistics) for the validation data group was 0.803 (95% CI, 0.738–0.867). When using the threshold value of 198, the sensitivity, specificity, positive predictive value, negative predictive value and concordance index were 75.7, 77.0, 83.2, 67.9%, and 0.52 respectively. Hosmer-Lemeshow test for evaluation of calibration showed that the Chi-square value is 11.074 (*P* = 0.135 > 0.05).

## Discussion

Literature review revealed that there were some reports on the survival prediction model of patients with EOC [6–10], while the recurrence prediction model of patients with EOC is relatively less [11]. In this study, the influencing factors related to the recurrence of EOC were screened out and evaluated by mathematical methods. And a predictive nomogram model of 3-year recurrence risk was established and verified externally. Comparison between the observed and expected prognosis suggested that this predicting model had good discrimination and calibration.

Many studies had confirmed that FIGO staging, histological grade, histological type, size of residual lesions, lymph node metastasis, serum CA125 level before treatment were associated with recurrence of EOC [11–16]. In our study, patients with advanced stage, serous carcinoma, high grade, lymph node metastasis and high serum CA125 level before treatment had relatively shorter RFI, which was consistent with the literature. Although Cox regression analysis confirmed that patients with no residual tumor had shorter RFI (*P* < 0.001) than the other patients, the LASSO regression didn’t put it into the nomogram model. This might be related to the inclusion criteria that all the patients should reach the status of CCR and only 10% of the patients had residual lesion size bigger than 1 cm, which may decrease the effect of residual lesion size on the recurrence risk.

The relationship between ER/PR expression and recurrence of epithelial ovarian cancer was controversial. A total of 2933 patients with epithelial ovarian cancer were included in Sieh’s study. It was found that ER-positive patients had a better prognosis in endometrioid cancer, while ER-positive patients in serous, mucinous and clear cell carcinomas had no significant correlation with prognosis. PR-positive patients in endometrioid and high-grade ovarian serous carcinomas had a better prognosis, while there was no significant correlation between PR positive expression and prognosis in patients with low-grade ovarian serous, mucinous carcinomas and clear cell carcinomas [17]. A meta-analysis of 35 studies showed that the disease-free survival (DFS) of patients with ER-positive EOC was better than that of patients with ER-negative EOC [18]. Therefore, the relationship between ER/PR expression and recurrence of ovarian cancer is not clear, and there are inconsistent conclusions among various studies. In our study, Cox regression analysis confirmed that patients with ER positive expression in tumor tissue had shorter RFI (HR, 1.713; 95% CI, 1.057–2.776; *P* = 0.029) than those with negative expression. However, given the literature review and high *P* value, we didn’t put this factor into the nomogram model.

At present, most of the studies related to the recurrence of EOC were still limited to obtaining Cox proportional risk model, which was complex and not convenient for clinical application [12–14]. In this study, the Cox proportional hazard model was transformed into a more intuitive and easy-to-calculate contour diagram model by using mathematical method and R software. When using this nomogram to predict the 3-year recurrence rate of EOC patients, the internal and external AUC (C statistics) obtained from ROC curve were 0.828 (95% CI, 0.764–0.884) and 0.803 (95% CI, 0.738–0.867) respectively, indicating that the model had a good distinction. Meanwhile, it could be seen in the calibration curve that although the actual red curve and the ideal black curve are not very different, the area divided by the blue curve representing 95% CI did not completely contain the ideal black curve, which showed that the calibration degree of the model was moderate. This might be related to the small sample size, resulting in greater fluctuation of the predicted value. Therefore, it is still necessary to increase the sample size and further adjust the model parameters to achieve better calibration in the future.

According to the RFI distribution of EOC patients, most relapsed patients would have recurrence within 3 years after primary treatment [1–5]. So in this study we aimed to stratify the 3-year recurrence risk of EOC patients by using the predictive nomogram model, as well as to individualize the treatment and follow-up plan according to the risk stratification. The result of external validation might ensure the transportability and generalizability of the nomogram. For EOC patients with high recurrence risk, aggressive maintenance therapy with targeted drugs, endocrinal therapy or immune medicine after chemotherapy may help to reduce the recurrence risk of such patients and improve their prognosis. And for patients at low risk of recurrence, we may reduce the frequency of follow-up appropriately and make individualized follow-up plan to lower the expenses in the first 3 years.

However, our study still had some limitation. This study was a retrospective cohort study and all the patients reached a status of CCR after primary treatment, which may both lead to the selection bias. The factors included were traditional clinicopathological factors, molecular markers (such as serum human epididymis protein 4, BRAC gene detection), targeted therapy, endocrine therapy, immunotherapy were not included in the scope of this study. The external verification results of the model indicated that a larger sampling and global multi-central recruitment was needed for model establishment and validation to ensure a better discriminative and calibration power. Prospective randomized controlled trials are still needed to prove the feasibility of layering treatment and follow-up plans according to recurrence risk.

## Conclusions

The nomogram constructed by FIGO staging, histological grade, histological type, lymph node metastasis and serum CA125 level before treatment could be used to predict the 3-year recurrence risk of patients who reach CCR after primary treatment. This nomogram with good discrimination and calibration might be useful for screening out the patients with high risk of recurrence.

## Data Availability

The datasets used and analysed during the current study are available from the corresponding author on reasonable request.

## Abbreviations

CCR: Clinical complete remission
ER: Estrogen receptor
PR: Progesterone receptor
CA125: Carbohydrate antigen 125
RFI: Recurrence-free interval
DFS: Disease-free survival
AUC: Area under curve
